# Supplementary material for: How effective is extracorporeal life support for patients with out-of-hospital cardiac arrest initiated at the emergency department? A systematic review and meta-analysis
Source: PLoS One. 2023 Nov 7;18(11):e0289054. doi: 10.1371/journal.pone.0289054 (PMC10629644; doi:10.1371/journal.pone.0289054)
Supplement: S1 Table — (DOCX) [file pone.0289054.s002.docx]

**Supplementary file**

Table S1. Electronic search terms

| Database | |
| --- | --- |
| PubMed | #1 ((((((((“heart arrest” (MeSH Terms)) OR (“Out-of-Hospital Cardiac Arrest” (MeSH Terms))) OR (cardiac arrest (Title/Abstract))) OR (cardiopulmonary resuscitation (Title/Abstract))) OR (CPR (Title/Abstract))) OR (resuscitation (Title/Abstract))) OR (out-of-hospital cardiac arrest (Title/Abstract))) OR (out-of-hospital (Title/Abstract)))  #2 ((((((“Extracorporeal Membrane Oxygenation” (MeSH Terms)) OR (“Extracorporeal Circulation” (MeSH Terms))) OR (extracorporeal (Title/Abstract))) OR (ECPR (Title/Abstract))) OR (ECLS (Title/Abstract))) OR (ECMO (Title/Abstract)))  #3 ((((((“Emergency Service, Hospital” (MeSH Terms)) OR (Emergency Department (Title/Abstract))) OR (Emergency Room (Title/Abstract))) OR (Emergency Unit (Title/Abstract))) OR (Emergency Physician (Title/Abstract))) OR (Emergency doctor (Title/Abstract)))  #1 AND (#2 OR #3) |
| EMBASE | #1 ('heart arrest'/exp OR 'arrest, heart' OR 'asystole' OR 'asystolia' OR 'asystoly' OR 'cardiac arrest' OR 'circulation arrest' OR 'circulatory arrest' OR 'heart arrest' OR 'heart arrest, induced' OR 'heart asystole' OR 'heart standstill' OR 'induced heart arrest')  #2 ('extracorporeal therapy'/exp OR 'extra corporal treatment' OR 'extra corporeal therapy' OR 'extra corporeal treatment' OR 'extracorporal therapy' OR 'extracorporal treatment' OR 'extracorporeal therapy' OR 'extracorporeal treatment')  #3 ('survival'/exp OR 'neurological outcome'/exp)  #4 ('emergency ward'/exp OR 'emergency department' OR 'emergency room' OR 'emergency unit' OR 'emergency ward')  #1 AND #2 AND #3 AND #4 |
| Web of Science | #1 heart arrest (All) or cardiac arrest (All) or cardiopulmonary resuscitation (All) or CPR (All) or resuscitation (All) OR out-of-hospital (All) or out-of-hospital cardiac arrest (All) or asystole (All) or shockable rhythm (All)  #2 extracorporeal life support (All) or extracorporeal membrane oxygenation (All) or extracorporeal (All) or ECLS (All) or ECMO (All) or extracorporeal cardiopulmonary resuscitation (All)  #3 emergency (All) or emergency department (All) or emergency ward (All) or emergency room (All) or emergency physician  #4 survival (All) or outcome (All)  #1 AND #2 AND #3 AND #4 |
| Cochrane Collaboration | extracorporeal AND arrest in Title Abstract Keyword - (Word variations have been searched) |
