# Supplementary material for: How effective is extracorporeal life support for patients with out-of-hospital cardiac arrest initiated at the emergency department? A systematic review and meta-analysis
Source: PLoS One. 2023 Nov 7;18(11):e0289054. doi: 10.1371/journal.pone.0289054 (PMC10629644; doi:10.1371/journal.pone.0289054)
Supplement: Attachment — Submitted filename: Response to Reviewers-R2.docx [file pone.0289054.s004.docx]

**Response to Reviewers**

We would like to express our sincere gratitude for the opportunity to revise our manuscript. Changes are marked with tracked changes and an unmarked version of the revised paper without tracked changes is provided.

**Editor Comments :
English language (syntax, grammar, correct choice of words, correct use of adjectives and adverbs) needs significant editing throughout the text. Professional assistance must be sought.**

**Response:** We sincerely appreciate the feedback provided. We have carefully considered your suggestions and taken necessary measures to significantly enhance the syntax, grammar, word choice, and appropriate usage of objectives and adverbs throughout the text. To ensure the utmost quality and professionalism in our writing, we engaged a specialized professional language editor. Their expertise allowed for meticulous revisions, greatly improving the document's readability and coherence. Changes are marked with tracked changes throughout the revised manuscript.
